# Deficiency of TTYH1 Expression Reduces the Migration and Invasion of U2OS Human Osteosarcoma Cells

**DOI:** 10.3390/life12040530

**Published:** 2022-04-03

**Authors:** Young-Sun Lee, Osung Kwon, Geuk-Rae Jeong, Junyeol Noh, Sung Eun Kim, Gwan-Su Yi, Eun Mi Hwang, Jae-Yong Park

**Affiliations:** 1 School of Biosystems and Biomedical Sciences, College of Health Sciences, Korea University, Seoul 02841, Korea; ssunny@korea.ac.kr (Y.-S.L.); osung@korea.ac.kr (O.K.); shinejkl@naver.com (G.-R.J.); nkjaing@naver.com (J.N.); sek19@korea.ac.kr (S.E.K.); 2 Center for Functional Connectomics, Korea Institute of Science and Technology (KIST), Seoul 02792, Korea; emhwang@kist.re.kr; 3 Department of Bio and Brain Engineering, Korea Advanced Institute of Science and Technology (KAIST), Daejeon 34141, Korea; gsyi@kaist.ac.kr

**Keywords:** osteosarcoma, TTYH1, invasion, migration, epithelial–mesenchymal transition

## Abstract

The *Tweety* *homolog* (TTYH) chloride channel family is involved in oncogenic processes including cell proliferation, invasion, and colonization of cancers. Among the TTYH family, TTYH1 is highly expressed in several cancer cells, such as glioma, breast, and gastric cancer cells. However, the role of TTYH1 in the progression of osteosarcoma remains unknown. Here, we report that deficient TTYH1 expression results in the inhibition of the migration and invasion of U2OS human osteosarcoma cells. We found that TTYH1 was endogenously expressed at both mRNA and protein levels in U2OS cells and that these channels were located at the plasma membrane of the cells. Moreover, we found that silencing of the TTYH1 with small interfering RNA (siRNA) resulted in a decrease in the migration and invasion of U2OS cells, while the proliferation of the cells was not affected. Additionally, treatment with TTYH1 siRNA significantly suppressed the mRNA expression of epithelial–mesenchymal transition (EMT)-regulated transcription factors such as Zinc E-Box Binding Homeobox 1 (ZEB1) and SNAIL. Most importantly, the expression of matrix metalloproteinase (MMP)-2, MPP-9, and N-cadherin was dramatically reduced following the silencing of TTYH1. Taken together, our findings suggest that silencing of TTYH1 expression reduces migration and invasion of U2OS cells and that TTYH1 may act as a potential molecular target for osteosarcoma treatment.

## 1. Introduction

Osteosarcoma, a type of bone cancer, is an aggressive, common malignant tumor in children and young adults [1,2]. Until now, osteosarcoma has mainly been treated through surgery, chemotherapy, and their combination treatment; however, their effects remain unsatisfactory, largely because patients with osteosarcoma exhibit lung metastasis and chemoresistance [3]. Therefore, the survival rates of patients with metastasis are significantly lower than those without metastasis. Despite many years of research, the detailed molecular mechanism of development and metastasis of osteosarcoma are still largely unknown.

Chloride channels have recently emerged as therapeutic targets for pharmacological agents in the migration, invasion, and metastasis of multiple human cancers [4]. These channels regulate electrical excitability, fluid transport, homeostasis, pH levels, and volume of cells, which are important for cancer progression [5,6]. Several studies have demonstrated that chloride channels are essential for regulating the proliferation, migration, invasion, metastasis, and malignant transformation of diverse cancer cells [7]. For example, TMEM16A, a calcium-activated chloride channel, is associated with cell proliferation, migration, invasion, and tumor growth in various cancers including glioblastoma [8], breast cancer [9], head and neck cancer [10], and gastric cancer [11]. Therefore, blocker or gene silencing of chloride channels results in the inhibition of migration and invasion capacity in cancer cells [12].

Among the chloride channels, the *tweety* (*tty* or *twe*) gene was identified in the flightless gene locus of *Drosophila melanogaster* [13,14]. Three *tweety homologs* (TTYH1, TTYH2, and TTYH3) have been identified in *Caenorhabditis elegans*, mice, macaques, and humans, and these channels have been shown to exhibit large-conductance chloride channel activity [14]. Members of the TTYH family contribute to cancer cell progression, including the cell cycle, invasion, and colonization of cancer metastasis in diverse cancers [15,16,17,18]. In particular, TTYH1 knockdown reduced invasiveness of glioma and decreased tumor size, and increased survival in mice [16]. Although TTYH1 is also expressed in several cancer cells including U2OS osteosarcoma cells (www.proteinatlas.org accessed on 10 November 2021), the roles of TTYH1 in cancer progression have not yet been examined.

In this study, we investigated the correlation between TTYH1 expression and cancer progression of U2OS human osteosarcoma cells. Silencing of TTYH1 inhibited the migration and invasion of the U2OS osteosarcoma cells and reduced the expression of epithelial-mesenchymal transition (EMT)-related factors such as SNAIL, Zinc E-Box Binding Homeobox 1 (ZEB1), matrix metalloproteinase 2 (MMP2), MMP9, and N-cadherin. Our findings suggest that TTYH1 could be used as a potential target for the treatment of osteosarcoma.

## 2. Materials and Methods

### 2.1. Cell Culture

U2OS, SaOS2, MG-63, and Fob1.19 cells were grown in Dulbecco’s modified Eagle’s medium (DMEM, HyClone, Logan, UT, USA) supplemented with 10% fetal bovine serum (Gibco, Thermo Fisher Scientific, Inc., Waltham, MA, USA) and 100 units/mL penicillin–streptomycin (Gibco, Thermo Fisher Scientific, Inc., Waltham, MA, USA) and incubated at 37 °C under 5% CO_2_.

### 2.2. Construction of Expression Vector and siRNA-Mediated TTYH1 Knockdown

The cDNA encoding the full-length human TTYH1 gene (GenBank Accession No. BC011347) was obtained using the RT-PCR-based gateway cloning method (Invitrogen, Thermo Fisher Scientific, Inc., Waltham, MA, USA). The construct was subcloned into pDEST-GFP-C using the gateway cloning method (Invitrogen, Thermo Fisher Scientific, Inc., Waltham, MA, USA). Transfection of the TTYH1 expression vector was performed using lipofectamine 2000 (Invitrogen, Thermo Fisher Scientific, Inc., Waltham, MA, USA) according to the manufacturer’s protocol.

Two different small interfering RNAs (siRNAs) specific for TTYH1 were used: siTTYH1-1, 5′-GCATTGGCATCGGTTTCTA-3′ and siTTYH1-2, 5′-GCTCCAATCCAGACCCTTA-3′. A negative control RNA was used (siCON, 5′-AATTCTCCGAACGTGTCAC-3′). U2OS cells were transfected with 100 nM siRNAs using INTERFERin^®^ (Polyplus, Illkirch, France) according to the manufacturer’s protocol for all experiments.

### 2.3. Reverse Transcription Polymerase Chain Reaction (RT-PCR) and Real-Time Quantitative RT-PCR (qRT-PCR)

Total RNA was extracted from four cultured cell clines, U2OS cells transfected with siRNAs, and SaOS2 cells transfected with TTYH1 expression vectors using an RNA purification kit (GeneAll Biotechnology, Seoul, Korea) following the manufacturer’s instructions. cDNAs were synthesized from 1 μg of total RNA, and reverse transcription was performed using iScript™ cDNA Synthesis Kit (BIO-RAD, Hercules, CA, USA) according to the manufacturer’s instructions. RT-PCR was performed with specific primer pairs (Table 1).

For qRT-PCR analysis, synthesized cDNAs were amplified using the SYBR Green mix (Enzo, New York, NY, USA) on an ABI Prism 7900 Sequence Detector. qRT-PCR was performed with specific primer pairs (Table 2). Melting curve analysis was added at the end of the program. Actin was used as a reference gene. The 2−ΔΔCt method was used to calculate fold changes in gene expression. All experiments were repeated at least three times.

### 2.4. Immunoblotting

U2OS cells transfected with siRNAs were extracted using RIPA butter (iNtRON Biotechnology Inc., Seongnam, Korea). Protein concentrations in the cells were determined using a bicinchoninic acid assay reagent kit (Pierce Chemical Co., Dallas, TX, USA) following the manufacturer’s instructions. The protein from each sample was separated by electrophoresis in 10% sodium dodecyl sulfate-polyacrylamide gels and transferred to polyvinylidene fluoride membranes. The membranes were blocked with 5% skim milk in TBST (Tris-buffered saline, 0.1% Tween 20) and incubated overnight at 4 °C with anti-TTYH1 (1:200; Cusabio technology LLC, Houston, TX, USA), anti-MMP2 (1:1000; Cell signaling technology, Danvers, MA, USA), anti-MMP9 (1:1000; Cell signaling), anti-N-cadherin (1:1000; Cell signaling), or anti-Actin (1:1000; Sigma Aldrich, St. Louis, MO, USA) antibodies. Then, the membranes were incubated with HRP-conjugated anti-mouse and anti-rabbit IgG antibodies at room temperature for 1 h. The intensity of the bands was quantified by densitometry (ImageJ, Bethesda, MD, USA) and normalized to the corresponding actin bands. All experiments were performed at least three times.

### 2.5. Immunofluorescence

U2OS cells transfected with siRNAs grown on coverslips were fixed with 4% paraformaldehyde for 20 min at room temperature and were permeabilized with 0.1% Triton X-100 in phosphate-buffered saline (PBS). The cells were blocked with 3% bovine serum albumin and then incubated overnight at 4 °C with anti-TTYH1 (1:200; Cusabio technology LLC, Houston, TX, USA) antibody. After rinsing in cold PBS, the cells were treated with DyLight 488-conjugated secondary antibodies (1:500; Jackson Labs, Harbor, ME, USA) for 1 h at room temperature. The cells were washed, mounted, and observed under an A1 confocal microscope (Nikon, Minato-ku, Tokyo, Japan).

### 2.6. Cell Viability Assay

The viability of cells was assessed using the cell counting kit (CCK, D-Plus™ cell counting kit-8, Dong-in LS, Anyang, Korea) according to the manufacturer’s instructions. U2OS cells were seeded in 96-well plates at a density of 5 × 10^3^ cells per well and incubated for 24 h. Cells were transfected with siRNAs and incubated for 48 h. After incubation, the CCK solution was added to each well, and the cells were incubated for 2 h at 37 °C under 5% CO_2_. Finally, the absorbance was measured at 450 nm wavelength using a spectrophotometer (Molecular Devices, San Jose, CA, USA).

### 2.7. Cell Invasion Assay

Cell invasion assay was performed using Transwell invasion chambers with 8.0 μm pore (Corning, Corning, NY, USA), according to the manufacturer’s instructions. Growth factor-reduced Matrigel (Corning, Corning, NY, USA) was used to coat the membrane. U2OS cells transfected with siRNAs and SaOS2 cells transfected with TTYH1 expression vectors were plated onto the Transwell membrane insert at a density of 5 × 10^4^ cells per well in 100 μL DMEM. The lower chambers were filled with 500 μL of DMEM. Transwells were incubated for 18 h to allow cell migration. Then, the cells from the upper side of the insert filter were completely removed using a cotton swab, and cells that had invaded through the coated membrane were fixed and stained using the Diff-Quick Stain kit (Sysmex, Kobe, Hyogo, Japan). For quantification, cells were counted under a microscope in three random fields at 20× magnification. All experiments were performed at least three times.

### 2.8. Cell Migration Assay

A total of 3.5 × 10^4^ cells per well U2OS cells transfected with siRNAs and 5 × 10^4^ cells per well SaOS2 cells transfected with TTYH1 expression vectors were plated onto SPLScar™ Block (SPL, Pocheon, Korea) in DMEM medium. After 24 h, the blocks were removed and incubated in a complete medium for 18 h or 24 h. Migrated cells were imaged. The remaining wound area was calculated relative to the initial wound area (at 0 h) and normalized to that reported for the siCON-transfected cells.

### 2.9. Statistical Analysis

All data were represented as mean ± standard error of the mean (SEM). Statistical analysis was performed using unpaired or paired Student’s t-test, with the significance level denoted by asterisks (n.s.; not significant, * *p* < 0.05, ** *p* < 0.01, or *** *p* < 0.001).

## 3. Results

### 3.1. TTYH1 Was Endogenously Expressed in U2OS Cells

**Figure 1:**
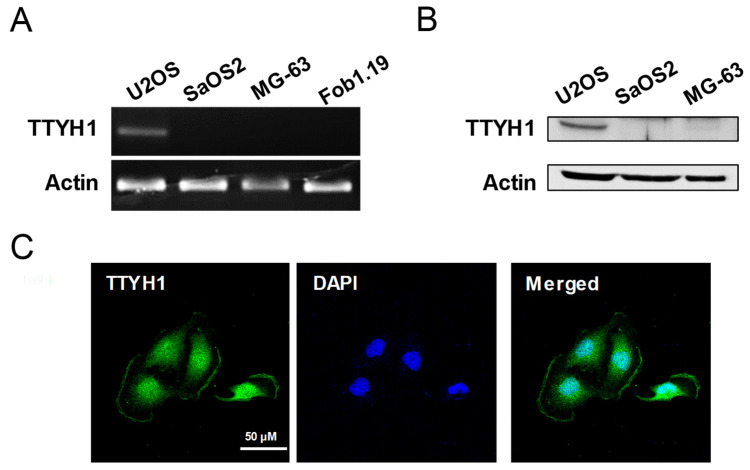
TTYH1 was endogenously expressed in U2OS cells. (**A**) RT-PCR was performed to analyze the mRNA expression levels of TTHY1 in three osteosarcoma cell lines (U2OS, SaOS2, and MG-63) and control Fob1.19 osteoblast cell line. (**B**) Western blotting was performed to analyze the protein expression levels of TTYH1 in U2OS, SaOS2, and MG-63 cells. (**C**) Representative immunofluorescence images showing TTYH1 expression in U2OS cells. Immunofluorescence staining was performed to detect subcellular localization of TTYH1 in U2OS cells.

**Figure 2:**
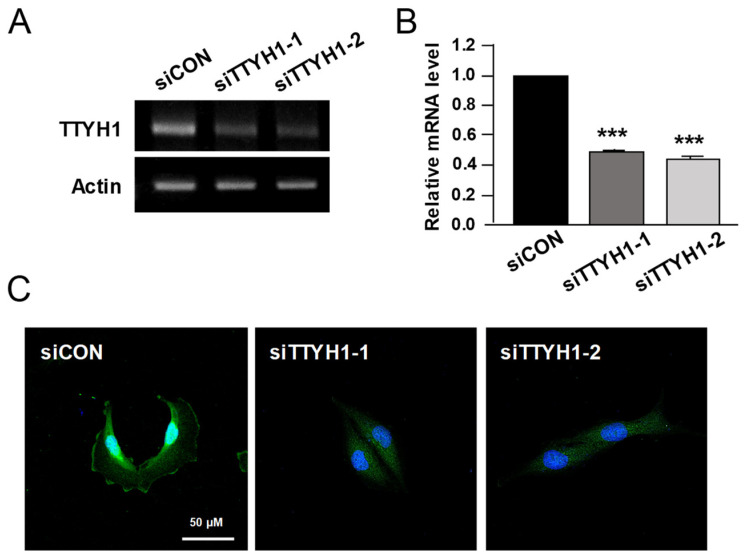
Validation of siTTYH1 effect in U2OS cells. (**A**) RT-PCR was performed to analyze the knockdown efficiency of siTTHY1 at the mRNA level in U2OS cells. (**B**) Normalization of mRNA levels of TTYH1 to those of Actin. (**C**) Representative immunofluorescence images showing TTYH1 expression after siCON, siTTYH1-1, or siTTYH1-2 transfection in U2OS cells. Data are represented as the mean ± standard deviation from three independent experiments. *** *p* < 0.001.

To explore whether TTYH1 expression is involved in the progression of osteosarcoma, we investigated the endogenous expression of TTYH1 at mRNA and protein levels using RT-PCR and Western blotting, respectively, in three different human osteosarcoma cell lines (U2OS, SaSO2, and MG-63). First, the results of RT-PCR showed that mRNA expression of TTYH1 was the highest in U2OS cells among three different osteosarcoma cell lines and control Fob1.19 osteoblast cells (Figure 1A). Similarly, the protein expression of TTYH1 was also the highest in U2OS cells (Figure 1B). Immunofluorescent staining data showed the cellular location of TTYH1 in U2OS cells (Figure 1C). These data indicate that TTYH1 is significantly expressed in human osteosarcoma U2OS cells. Next, to determine whether TTYH1 expression levels also correlate with the cancerous properties of osteosarcoma cells, we used two different siRNAs constructs against TTYH1 (siTTYH1) and examined the gene silencing efficiency of these siRNAs in osteosarcoma cells. We investigated whether TTYH1 expression levels correlate with the increase in the cancer progression-related properties of U2OS cells. The following siRNA constructs were designed for TTYH1-specific knockdown in U2OS cells: siRNA control (siCON), siRNA TTYH1-1 (siTTYH1-1), or siRNA TTYH1-2 (siTTYH1-2). U2OS cells were transfected with these siRNAs for 48 h, and the silencing efficiency of siTTYH1-1 and siTTYH1-2 in U2OS cells was analyzed by RT-PCR and immunofluorescent staining assay (Figure 2A–C). We observed that the mRNA level of TTYH1 was significantly reduced by siTTYH1-1 and siTTYH1-2 by 50~60% in U2OS cells (Figure 2A,B). Results from the immunofluorescent staining assay revealed that TTYH1 was expressed in siCON-transfected cells and that TTYH1 expression was successfully reduced by siTTYH1-1 and siTTYH1-2 (Figure 2C).

### 3.2. Silencing of TTYH1 Gene Expression Inhibited the Migration and Invasion of U2OS Cells

**Figure 3:**
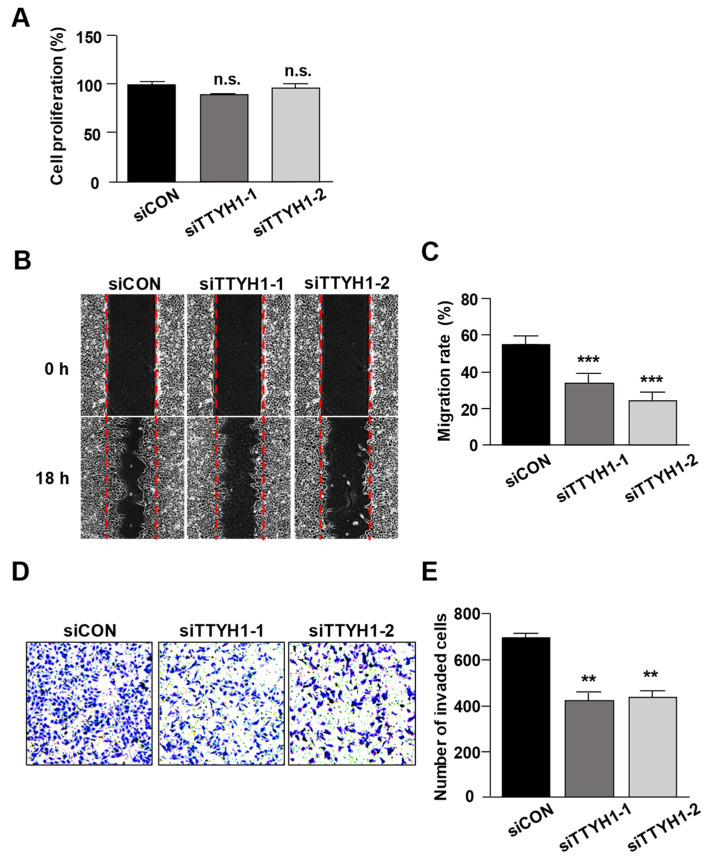
Silencing of TTYH1 gene expression inhibited the migration and invasion, but not the proliferation, of U2OS cells. (**A**) Cell Counting Kit-8 (CCK-8) assay was performed to determine the proliferation of U2OS cells after siCON, siTTYH1-1, or siTTYH1-2 transfection. (**B**,**C**) Cell migration assay was performed to evaluate the migration ability of U2OS cells after siCON, siTTYH1-1, or siTTYH1-2 transfection. (**D**,**E**) Transwell invasion assay was performed to evaluate the invasion abilities of U2OS cells after siCON, siTTYH1-1, or siTTYH1-2 transfection. Data are represented as the mean ± standard deviation from three independent experiments. Not significant (n.s.), ** *p* < 0.01 or *** *p* < 0.001.

The effect of TTYH1 knockdown on the viability of U2OS cells was measured by the CCK assay. Results showed that TTYH1 knockdown did not affect the proliferation of U2OS cells (Figure 3A). Next, we investigated whether TTYH1 enhances the migration ability of U2OS cells. The results revealed that TTYH1 knockdown significantly reduced the number of migrated cells by more than 50% compared to that in siCON-transfected cells (Figure 3B,C). We also assessed the invasion ability of U2OS cells following TTYH1 knockdown. Our results showed that the number of invaded cells was markedly decreased by 40% after transfection with siTTYH1s compared with that of the control in U2OS cells (Figure 3D,E). These results indicated that TTYH1 contributes to the migration and invasion of U2OS cells.

### 3.3. Overexpression of TTYH1 Promoted the Cell Migration and Invasion Abilities of a TTYH1 Non-Expressed Osteosarcoma Cell, SaOS2 Cell

**Figure 4:**
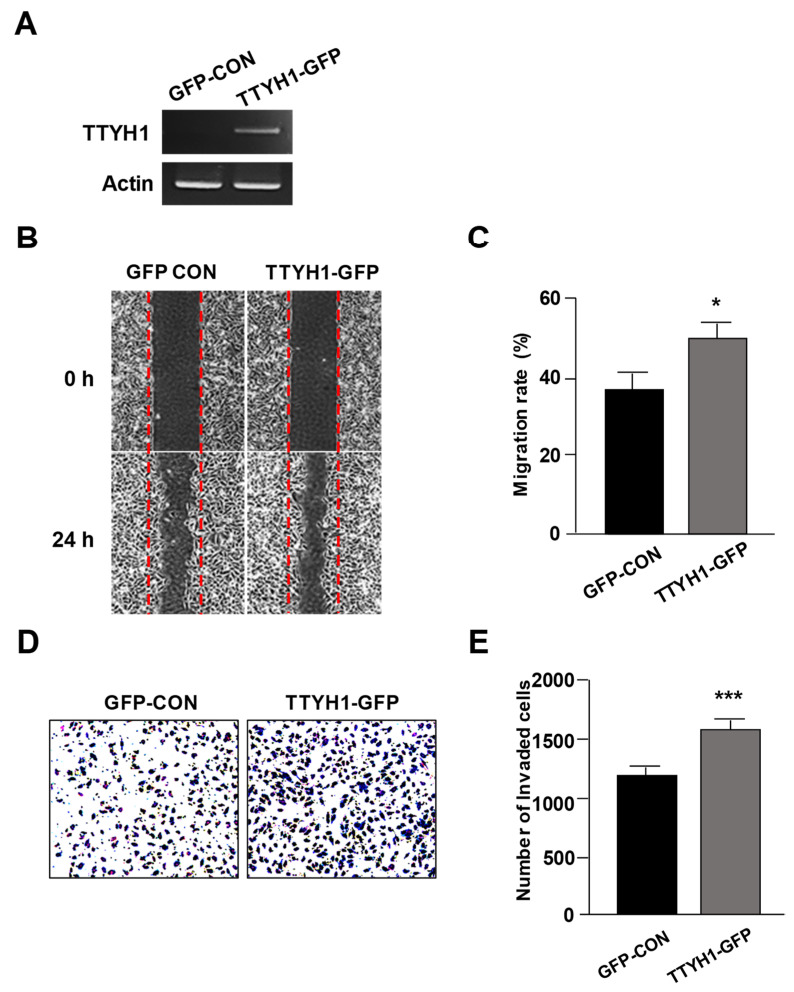
Overexpression of TTYH1 increased the migration and invasion of SaOS2 cells. (**A**) RT-PCR against TTYH1, (**B**,**C**) cell migration assay, and (**D**,**E**) cell invasion assay of SaOS2 cells after GFP-CON (GFP–control) or TTYH1–GFP transfection. Data are represented as the mean ± standard deviation from three independent experiments. * *p* < 0.05 or *** *p* < 0.001.

As shown in Figure 1, TTYH1 was found to be overexpressed in U2OS cells, but not in MG-63 and SaOS2 cells. To study the effects of TTYH1 overexpression in osteosarcoma cell lines, we transfected TTYH1-GFP into SaOS2 cells and confirmed TTYH1 expression using RT-PCR analysis (Figure 4A). As expected, overexpression of TTYH1 significantly increased cell migration (Figure 4B,C). In addition, we found that overexpression of TTYH1 increased cell invasiveness of SaOS2 cells (Figure 4D,E). Overall, these data suggested that TTYH1 can play an important role in the cell migration and invasion of osteosarcoma cells.

### 3.4. Silencing of the TTYH1 Gene Interfered with the Expression of Epithelial–Mesenchymal (EMT)-Related Factors

**Figure 5:**
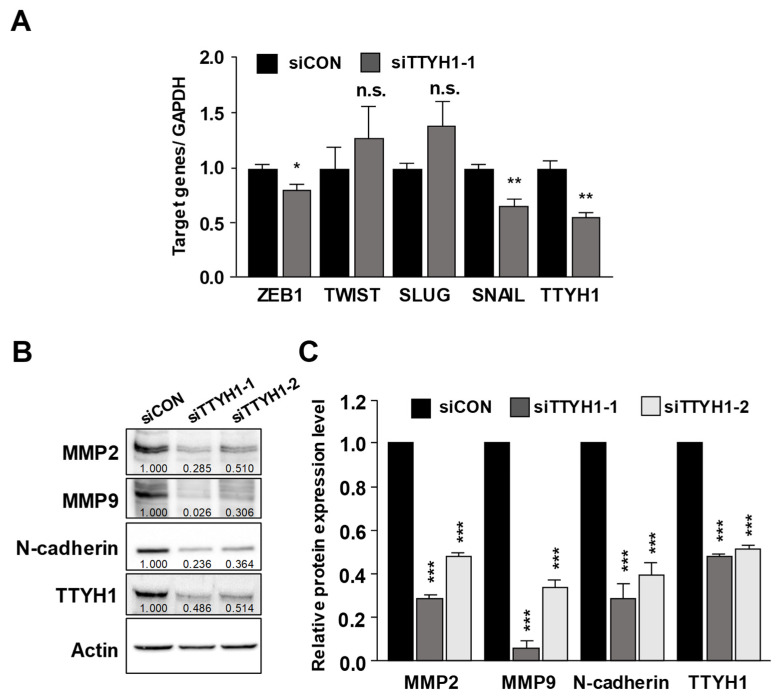
Silencing of the TTYH1 gene interfered with the expression of epithelial–mesenchymal (EMT)-related factors. (**A**) Quantitative real-time PCR (qRT-PCR) was performed to evaluate the mRNA expression levels of ZEB1, TWIST, SLUG, and SNAIL in siTTYH1-1-transfected U2OS cells. The mRNA levels of ZEB1, TWIST, SLUG, and SNAIL were normalized to those of GAPDH. (**B**,**C**) Western blotting was performed to analyze the expression of MMP2, MMP9, N-rin, and TTYH1 proteins in siRNAs-transfected U2OS cells. The expression of MMP2, MMP9, N-cadherin, and TTYH1 was normalized to that of actin. Data are represented as the mean ± standard deviation from three independent experiments. Not significant (n.s.). * *p* < 0.05, ** *p* < 0.01, or *** *p* < 0.001.

Epithelial-to-mesenchymal transition (EMT) is involved in the motility and invasiveness of cancerous cells [19]. On the basis of the effectiveness of TTYH1 knockdown on the migration and invasion of U2OS cells (Figure 3), we examined if TTYH1 is involved in the expression of EMT-related transcription factors, such as ZEB1, TWIST, SLUG, and SNAIL, using qRT-PCR (Figure 5A). Results of qRT-PCR showed that the expression of ZEB1 and SNAIL was significantly reduced by 20% and 30%, respectively, after siTTYH1-1 transfection. Additionally, we observed that TTYH1 effectively regulated the protein expression levels of MMP-2/9 and N-cadherin, which are involved in the migration and invasion of cancer cells. Results of Western blotting showed that silencing of TTYH1 by siRNAs markedly suppressed the protein levels of MMP-2/9 and N-cadherin (Figure 5B,C). These data indicated that silencing of TTYH1 inhibited the migration and invasion of U2OS cells via ZEB1, SNAIL, MMP2/9, and N-cadherin. Thus, our data indicated that TTYH1 was endogenously expressed and that knockdown of TTYH1 significantly reduced the migration and invasion of human osteosarcoma U2OS cells. Furthermore, we demonstrated that TTYH1 promoted the progression of osteosarcoma cancer by regulating the expression of EMT-related transcription factors and MMPs.

## 4. Discussion

Chloride channels, which regulate the migration, invasion, and metastasis of diverse cancer cells, have emerged as promising therapeutic targets [4]. Among chloride channels, the TTYH family is involved in several processes, including cell division, cell adhesion, regulation of calcium activity, and cancer progression. Among the three members of the TTYH family, TTYH1 has been extensively studied. In neuronal stem cells, TTYH1 is highly expressed and is associated with cell proliferation via the Notch signaling pathway in the development of the mammalian brain [20]. Interestingly, TTYH1 drives tumor microtubule-mediated colonization of glioma cells [15] and serves as volume-regulated anion channels (VRACs) in several cancer cells such as SNU-601 gastric cancer cells and HepG2 liver cancer cells [16]. Although TTYH1 is also expressed in several cancer cells (www.proteinatlas.org accessed on 10 November 2021), the roles of TTYH1 in cancer cells are largely unknown.

Here, we found that TTYH1 is highly expressed in human osteosarcoma U2OS cells (Figure 1) and that the knockdown of TTYH1 did not affect the proliferation of U2OS cells but significantly suppressed the migration and invasion of U2OS cells (Figure 2 and Figure 3). Overexpression of TTYH1 is sufficient to promote the ability of cell migration and invasion in the TTYH1 non-expressed SaOS2 osteosarcoma cell (Figure 4). We also observed that the expression of SNAIL and ZEB1 was significantly downregulated after TTYH1 silencing (Figure 5A). Additionally, we demonstrated that the expression of MMP2/9 and N-cadherin was decreased in U2OS cells after TTYH1 downregulation (Figure 5B,C). The effects of TTYH1 on U2OS cells were consistent with our previous study, which showed that TTYH2 regulated the invasion and migration of the same cells [17]. In addition, we showed that TTYH1 and TTYH2 act as VRACs, which are involved in the regulation of cell volume, migration, and death [21,22]. On the basis of our previous and current studies, we believe that TTYH1 and TTYH2 can serve as potential therapeutic targets in osteosarcoma.

Patients with osteosarcoma to a high degree exhibit malignancy that tends to metastasize to other organs, remaining a fatal complication after treatment [3,23,24]. The formation and development of metastasis occur after the migration and invasion of cancer cells [25]. It has been emphasized that EMT is significantly involved in cancer invasion and metastasis of osteosarcoma [26,27,28]. EMT is regulated by E-cadherin, N-cadherin, vimentin, fibronectin, MMP-2, MMP-7, and MMP-9 [29]. The expression of these proteins is induced by transcription factors such as ZEB1/2, SNAIL, SLUG, and TWIST [30]. As shown in Figure 5, deficiency of TTYH1 results in downregulation of EMT-related pathways and expression of transcription factors, implying that TTYH1 could be a potential target for the treatment of osteosarcoma. EMT transcription factors such as ZEB1/2, SNAIL, and SLUG are associated with the Notch signaling pathway in various cancer cells [31]. In addition, recent studies reported Notch signaling promotes metastasis of osteosarcoma cells including the U2OS cell line, and blocking the Notch signaling pathway attenuates the osteosarcoma EMT-like phenotype [32,33]. Interestingly, recent studies suggested TTYH1 is involved in Notch signaling [20,34]. When a γ-secretase inhibitor, a downregulator of Notch signaling, is treated in mouse neurospheres, mRNAs and proteins of TTYH1 are reduced [34]. In contrast, overexpression of TTYH1 increases Notch intracellular domain (NICD) activation in neural stem cells through the increased maturation of γ-secretase complex by the degradation of retention in endoplasmic reticulum-sorting receptor 1 (RER1). Therefore, although it is unclear as to whether TTYH1 acts downstream or upstream (or both) of NICD activation, the Notch signaling pathway may serve as an important regulating mechanism in the crosstalk between TTYH1 silencing and downregulated expression of EMT transcription factors, as shown in Figure 5. Further studies will be required to examine how TTYH1 silencing is involved in the reduction of EMT phenotype in osteosarcoma cells. In addition, the clinical significance of TTYH1 in osteosarcoma is still unclear, although our data showed involvement of TTYH1 in the migration and invasion of osteosarcoma cell lines. Therefore, the potential role of TTYH1 in the clinical evidence associated with the progression of osteosarcoma should be examined in future studies.

Interestingly, two individual cryo-EM studies reported TTYH proteins are not pore-forming subunits of anion channels cryo-EM structures and suggested their function may involve interactions with other components such as lipid-like compounds residing in the membrane, hydrophobic molecules, or additional protein partners [35,36]. On the basis of these reports, it seems that unknown functioning of TTYH1 as a non-conducting protein might be important to understanding the roles of TTYH1 in the progression of osteosarcoma. Previously, a biotin-streptavidin pulldown assay identified diverse TTYH1 binding proteins including cytoskeletal proteins and proteins localized in the endoplasmic reticulum or Golgi apparatus [37]. In addition, overexpression of TTYH1 induced growth of filopodia, and endogenous TTYH1 is localized to neurites of hippocampal neurons and to extending processes of astrocytes [37,38,39]. Therefore, it is plausible that TTYH1 might be involved in diverse biological processes such as cell morphology, cell migration, and cell adhesion. Functional roles of TTYH1 with binding proteins in osteosarcoma should be further studied in future studies.

Taken together, these results revealed that silencing of TTYH1 regulates the migration and invasion of U2OS osteosarcoma cells and reduces the expression of MMP2/9, N-cadherin, ZEB, and SNAIL. In particular, the elucidation of the functions of TTYH1 in U2OS cells could lead to the development of improved treatment strategies for osteosarcoma, presenting with high metastasis rates and limited therapeutic options.

## Figures and Tables

**Table 1:** The primers used in RT-PCR.

Gene	Primer	Sequence
TTYH1	Forward	5′-CGAGAAGCTGTGCCTCAGTT-3′
	Reverse	5′-TGCAGACAGCAGGGAGAAGA-3′
Actin	Forward	5′-CACCAACTGGGACGACAT-3′
	Reverse	5′-ACAGCCTGGATAGCAACG-3′

**Table 2:** The primers used in qRT-PCR.

Gene	Primer	Sequence
TTYH1	Forward	5′-ACAGTGAGACCAGTGATGGG-3′
	Reverse	5′-GGTCAATGGTGCTGAGTGTG-3′
SNAIL	Forward	5′-GGACCCACACTGGCGAGAAG-3′
	Reverse	5′-ATTCGGGAGAAGGTCCGAGC-3′
SLUG	Forward	5′-TGTGACAAGGAATATGTGAGCC-3′
	Reverse	5′-TGAGCCCTCAGATTTGACCTG-3′
ZEB1	Forward	5′-CAGCCCTGCAGTCCAAGAAC-3′
	Reverse	5′-TTGTCTTTCATCCTGATTTCCATTT-3′
TWIST	Forward	5′-CGCCCCGCTCTTCTCCTCT-3′
	Reverse	5′-GACTGTCCATTTTCTCCTTCTCTG-3′
GAPDH	Forward	5′-GTCTTCACCACCATGGAGAA-3′
	Reverse	5′-GCATGGACTGTGGTCATGAG-3′
Actin	Forward	5′-CACCAACTGGGACGACAT-3′
	Reverse	5′-ACAGCCTGGATAGCAACG-3′

## Data Availability

Not applicable.
